# Prognostic Significance of CIP2A in Esophagogastric Junction Adenocarcinoma: A Study of 65 Patients and a Meta-Analysis

**DOI:** 10.1155/2019/2312439

**Published:** 2019-08-22

**Authors:** Yanhong Li, Mei Wang, Xueping Zhu, Xu Cao, Yi Wu, Fang Fang

**Affiliations:** ^1^Institute of Pediatric Research, Children's Hospital of Soochow University, Suzhou, 215025 Jiangsu, China; ^2^Department of Nephrology, Children's Hospital of Soochow University, Suzhou, 215025 Jiangsu, China; ^3^Department of Pharmacy, Children's Hospital of Soochow University, Suzhou, 215025 Jiangsu, China; ^4^Department of Neonatology, Children's Hospital of Soochow University, Suzhou 215025, China; ^5^Department of Surgery, Children's Hospital of Soochow University, Suzhou, 215025 Jiangsu, China; ^6^Department of Pathology, Children's Hospital of Soochow University, Suzhou, 215025 Jiangsu, China

## Abstract

**Background:**

The expression of the cancerous inhibitor protein phosphatase 2A (CIP2A) appears to be predictive of the prognosis of various solid tumors. However, the association between this protein and the risk of esophagogastric junction adenocarcinoma (EGJA) remains unclear. We investigated CIP2A expression and its clinical significance in EGJA and conducted a meta-analysis to explore the relationship between CIP2A and the prognosis of patients with solid tumors.

**Methods:**

Immunohistochemistry (IHC) was performed to detect the expression of CIP2A in EGJA. Kaplan-Meier estimation, Cox analysis, and ROC curves were performed to analyze the survival of patients and the prognostic factors. In the meta-analysis, we searched relevant publications in several widely used databases and used 15 studies (2348 patients).

**Results:**

IHC demonstrated that CIP2A was elevated in EGJA and correlated with poor survival as an independent indicator. It could forecast the survival more precisely when combined with the grade, which is another independent prognosis marker of EGJA. Meta-analysis demonstrated that the associations between the expression of CIP2A and the prognosis were detected for overall survival (HR = 1.98, 95%CI = 1.69‐2.32), disease-specific survival (HR = 1.72, 95%CI = 1.50‐1.97), and time to tumor progression (pooled HR = 1.95, 95%CI = 1.56‐2.43).

**Conclusion:**

High expression of CIP2A was a poor indicator of the prognosis of EGJA, and CIP2A may be a new biomarker for the diagnosis and treatment of EGJA. The meta-analysis suggested that CIP2A expression can be a predictive marker of overall survival, disease-specific survival, and time to tumor progression in patients with solid tumors.

## 1. Introduction

Esophageal squamous cell carcinoma (ESCC) and gastric carcinoma (GC) are common tumors and the leading causes of cancer-related deaths worldwide [1]. Esophagogastric junction adenocarcinoma (EGJA) is considered a unique clinical malignancy with different etiology, clinicopathological features, and biological behaviors than ESCC and GC. EGJA is defined as a cancer across the esophagogastric junction line, including distal esophageal adenocarcinoma and proximal gastric cancer [2, 3]. Chronic gastroesophageal reflux disease is a strong risk factor for EGJA and leads to intestinal metaplasia (Barrett's esophagus) [4]. Interestingly, *Helicobacter pylori* infection, a risk factor for gastric cancer, is considered a protective factor for EGJA for its prevention of reflux esophagitis and Barrett's esophagus [5]. The treatment strategy for EGJA is complex because of the anatomical location of the esophagogastric junction. The incidence of EGJA has shown an increasing trend over the past few decades with a poor prognosis [6, 7]. There is therefore an urgent need to reveal novel molecular markers to provide new strategies for EGJA treatment and improve patient outcomes.

Cancerous inhibitor protein phosphatase 2A (CIP2A) is an oncoprotein [8]. It is encoded by *KIAA1524* and functions through an “oncogenic nexus” [9]. The “oncogenic nexus” consists of various functions of CIP2A, including the inhibitory effects on protein phosphatase 2A, interactions with MYC protein, and effects on the mechanistic target rapamycin kinase (MTOR) [10]. Although CIP2A has been suggested to influence the prognosis of various cancer types [11–13], the role of CIP2A in EGJA remains unknown.

This study investigated the clinical significance of CIP2A in EGJA by detecting the expression of CIP2A in EGJA and paracancer tissues, providing a basis for clinical diagnosis, prognosis, and treatment guidance. Furthermore, we investigated the relationships between CIP2A and solid cancer prognosis systematically and statistically based on previous studies that focused on this relationship. A total of 15 studies with relatively large sample sizes (2348 patients total) were selected [14–28], and a meta-analysis was conducted to provide a more reliable assessment of the relationship between CIP2A and solid cancer prognosis.

## 2. Materials and Methods

### 2.1. Specimen Collection

The clinical tissue microarray of esophagogastric junction adenocarcinoma (HGEj-Ade130Sur-01) was purchased from Outdo Biotech Co. (Shanghai, China). Hematoxylin- and eosin-stained (HE) sections were used to determine the pathological and cytological diagnosis by pathologists based on the WHO Classification of Tumors. This study was approved by the Medical Ethical Committee of Children's Hospital of Soochow University.

### 2.2. Immunohistochemistry

According to the manufacturer's instructions, a catalyzed signal amplification system kit (Boster, Wuhan, China) microarray was deparaffinized, rehydrated, and the antigens retrieved. Sections were then incubated with the primary antibody (Anti-CIP2A, Santa Cruz Biotechnology, Santa Cruz, CA, USA) at a dilution of 1 : 100. The secondary antibody, developed using DAB, was counterstained with hematoxylin and observed under a microscope.

To determine the immunoreactivity scores (IRSs), the microarray was scored and evaluated by 3 investigators. Positive cells exhibited yellow or yellow-brown granules in the cytoplasm and/or nucleus. The staining intensity was scored as follows: colorless (no staining) was 0 points, light yellow (weak staining) was 1 point, yellow-brown (moderate staining) was 2 points, and brown (strong staining) was 3 points. The percentage of positive cells was scored as follows: ≤25% positive cells were scored as 1 point, between 26% and 50% positive cells were scored as 2 points, between 51% and 75% positive cells were scored as 3 points, and >75% positive cells were scored as 4 points; IRS = staining intensity × positive rate. The immunoreactivity was analyzed as negative (score = 0), weak positive (0 < score ≤ 6), and strong positive (6 < scores ≤ 12). In the final analysis, IRSs ≤ 6 were defined as CIP2A low expression, while IRSs > 6 were defined as CIP2A high expression [29].

### 2.3. Statistical Analyses

Wilcoxon's or Fisher's exact tests were applied to determine the strength of the association between the categorical variables. Kaplan-Meier analysis was performed to analyze the survival of patients, and the log-rank test was used to compare the survival curves. The Cox regression model was used to analyze the hazard ratio (HR) of the prognostic factors. The area under the curve (AUC) of the receiver operating characteristics (ROC) curve was used to quantitatively evaluate the specificity, sensitivity, and accuracy of the prognostic factors [30]. All of the statistical analyses were performed using SPSS 20.0 (SPSS Inc., Chicago, IL, USA). *P* value < 0.05 was considered statistically significant.

### 2.4. Literature Search, Selection, and Data Collection

In this study, we searched papers published before August 7, 2017, using the keywords “cell proliferation regulating inhibitor of protein phosphatase 2A”/“CIP2A”/“p90”/“KIAA1524”, “tumor”/“cancer”/“carcinoma”/“neoplasm”, and “prognosis”/“survival”/“death”/“mortality” in the PubMed and Web of Science databases independently. Papers obtained were further selected for the meta-analysis, and the selection criteria were (a) full-text English-language study, (b) sufficient and clear data for individual HR and 95% CI extraction, and (c) studies sharing the same sample of patients were compared and the most complete were included in the meta-analysis.

In this study, 3 researchers extracted data from eligible papers independently. Data were composed of the first author, publication year, country of origin of patients, tumor type, number of the patients, stage or grade of tumor, detection method, ratio of high CIP2A expression, cutoff value, median follow-up months, outcome endpoints, method of survival analysis, and HR and 95% confidence interval (CI) for high CIP2A expression group vs. the low CIP2A expression group. Multivariate HR and 95% CI were selected when univariate and multivariate results were both reported in an individual study. The final data collection was determined by agreement between the three investigators.

### 2.5. Meta-Analysis Methods

A meta-analysis of outcomes was performed in order to explore the association between CIP2A and solid tumor prognosis using the data collected from each eligible paper with the methods described in our previous study [31]. We used Stata 14.0 (Stata Corporation, College Station, TX, USA) to conduct the statistical analysis. Outcome endpoints recurrence-free survival (RFS) and disease-free survival (DFS), which are similar in meaning, were combined and a unified prognostic parameter, time to tumor progression (TTP), was used in the meta-analysis. We calculated the pooled HRs and 95% CIs for the 3 outcome endpoints overall survival (OS), disease-specific survival (DSS), and TTP. During calculation, fixed effects or random effects model was chosen according to the heterogeneity test. In the heterogeneity test, the *χ*^2^-based *Q*-test was performed [32]. If the *Q*-test reported a *P* value higher than 0.05, the fixed effects model was chosen [33]; otherwise, we used the random effects model [34].

We also tested publication bias by Begg's funnel plot and Egger's test [35]. When the funnel plot is asymmetric and *P* value is lower than 0.05 in Egger's test, publication bias is likely to exist.

## 3. Results

### 3.1. CIP2A Is Upregulated in Human EGJA and Correlates with Poor Survival

**Figure 1:**
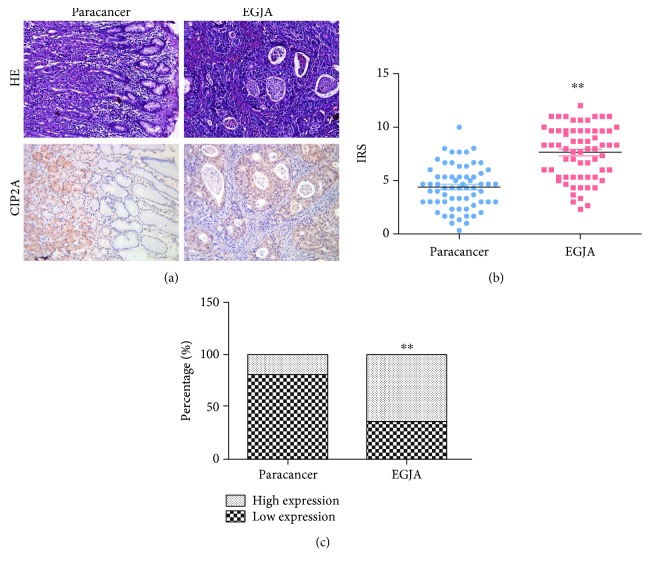
The expression of CIP2A in EGJA. (a) Hematoxylin-eosin (HE, upper) and immunohistochemistry (IHC, lower) staining of paracancer (left) and esophagogastric junction adenocarcinoma (EGJA, right). (b) Immunoreactivity score (IRS) comparison of CIP2A between paracancer and EGJA (Wilcoxon's test, ^∗∗^*P* < 0.01). (c) Expression percentage comparison of CIP2A between paracancer and EGJA (Fisher's exact test, ^∗∗^*P* < 0.01).

The expression of CIP2A in EGJA has not been reported in the current literature. To investigate the clinical significance, we investigated the expression of CIP2A in EGJA and paracancers by immunohistochemistry. As shown in Figure 1(a), CIP2A expression was mainly localized to the cytoplasm. In EGJA tissues, CIP2A was extensively expressed in cancer cells while in paracancers CIP2A was restricted to the fundic glands. Then, we scored the immunoreactivity and the IRSs were 4.36 ± 2.02 and 7.62 ± 2.48 in the paracancer and EGJA groups, respectively. Comparing with paracancer tissues, the IRSs indicated that CIP2A was highly expressed in EGJA cancer cells (Figure 1(b)). In addition, the expression level of CIP2A was divided into high expression and low expression groups according to the score. The expression rate of CIP2A in EGJA (64.6%) was significantly higher than that in paracancers (20.0%) (Figure 1(c)).

We further investigated the correlations of CIP2A expression with the clinicopathologic parameters in EGJA. The microarray contained 65 patients with EGJA, including 51 males and 14 females, with a median age of 71 years (44-84 years). Grades based on the WHO Classification of Tumors were divided into two groups, including low grade (39 cases) and high grade (26 cases). According to the TNM stage classification, 20 and 45 patients were classified as early (I-II) and late (III-IV) stages, respectively. A total of 69.2% (45 cases) of patients had lymph node metastasis, and 3.1% (2 cases) had distant metastasis. As the distant metastasis group contained very low case numbers, it was excluded in further analysis. Correlation analysis demonstrated no significant correlation between CIP2A expression and the clinicopathologic parameters (Table 1), indicating that CIP2A may be an independent factor of these parameters.

**Figure 2:**
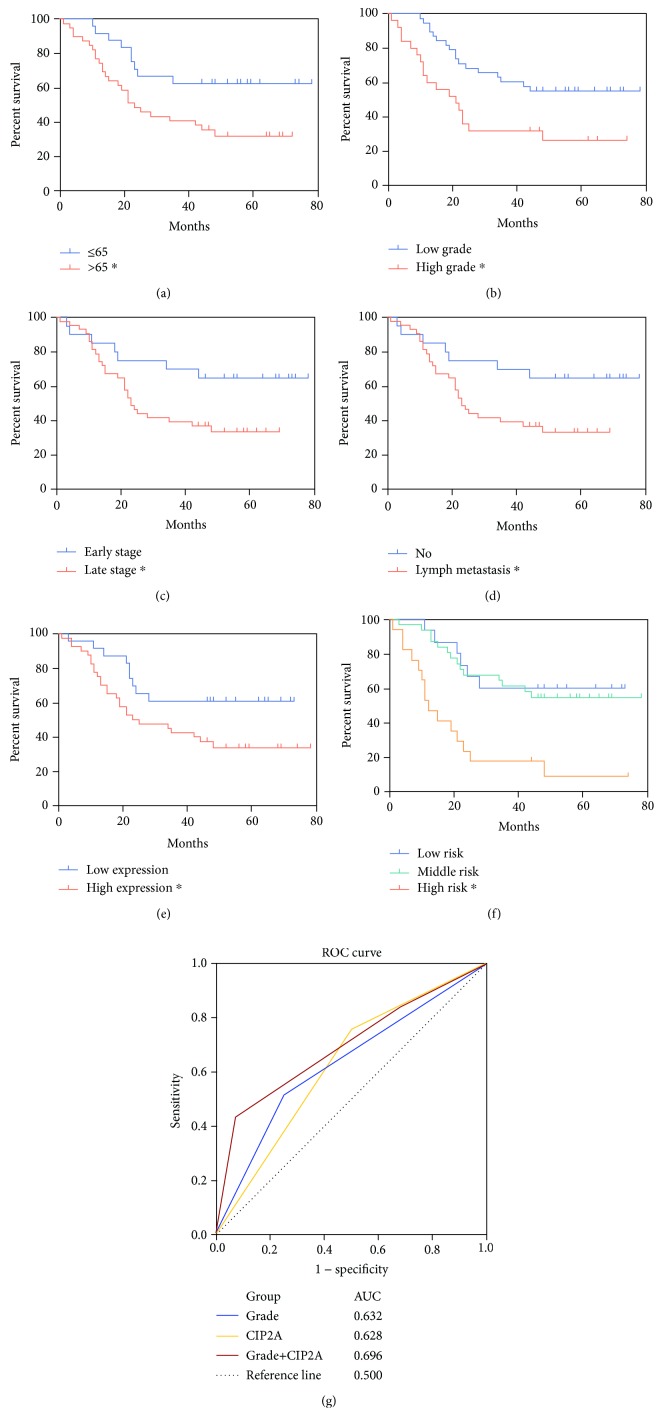
Survival analysis of clinicopathologic parameters and CIP2A expression. Kaplan-Meier analysis of survival based on (a) age, (b) grade, (c) stage, (d) lymph metastasis, (e) CIP2A expression, and (f) combination of grade and CIP2A expression (log-rank test, ^∗^*P* < 0.05). Low risk: low grade and low CIP2A expression; middle risk: low grade and high CIP2A expression/high grade and low CIP2A expression; high risk: high grade and high CIP2A expression. (g) ROC analysis of survival prediction power. The area under the curve (AUC) value was calculated from the ROC curve.

The median follow-up time of 65 EGJA patients in the microarray was 34.0 months, and the disease-caused death was rated at 56.9% of total (37/65). Fisher's exact test revealed that the death of the EGJA patients significantly correlated with age, grade, TNM stage, lymph metastasis, and CIP2A expression level (Supplementary [Supplementary-material supplementary-material-1]). Kaplan-Meier analysis further revealed that advanced age, high grade, late stage, lymph metastasis, and high expression of CIP2A were associated with poor patient prognosis (Figure 2). A univariate Cox regression analysis of the clinicopathologic parameters and CIP2A expression with patient survival was also performed, and the variables except for gender were identified as candidate prognostic factors for EGJA patients (Table 2). To assess whether the CIP2A expression for the prediction of survival was independent of these clinicopathologic parameters, we further performed a multivariable Cox regression analysis. The results demonstrated that the prognostic power of CIP2A was independent of these parameters (Table 2). In addition, the pathologic grade classification also independently affected patient survival (Table 2).

Combining CIP2A with the grade further subdivided the patients into three separate groups (Figure 2(f)). A Kaplan-Meier analysis of the individual patient groups demonstrated that combining these two factors could more precisely forecast survival (Figure 2(f)). The log-rank test showed that the high-risk group had shorter survival while the middle- and low-risk groups had longer survival. To compare the sensitivity and specificity of survival prediction between CIP2A and the grade, we performed a ROC analysis to calculate the area under the ROC curve (AUC), assuming that a larger AUC implies a better model for prediction. In the results, the combination model with a larger AUC indicated a better predictive ability than CIP2A or the grade alone (Figure 2(g)).

### 3.2. Meta-Analysis Results

**Figure 3:**
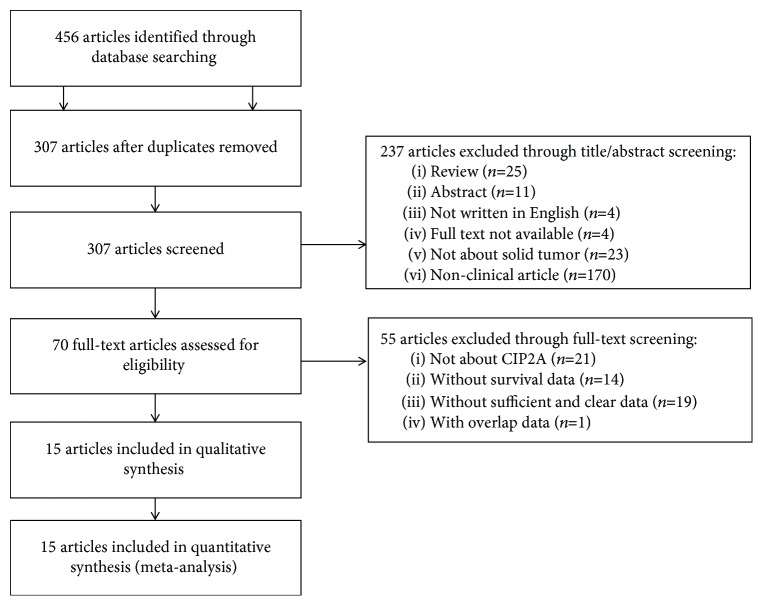
Flow chart of study selection.

Through searching and selection, a final cohort of 15 studies [14–28] was collected for quantitative synthesis (Figure 3). All 15 studies were studies of various ethnicities (12 studies of Asians and 3 studies of Caucasians) and cancer types (2 studies of hepatocellular carcinoma, 2 studies of renal cell carcinoma, and 11 studies of other cancer types).  Table 3 presents the data of the 15 studies. Of the studies, 8 focused on OS, 2 focused on DSS, and the other 5 studied two outcome endpoints. Among the 15 studies, 12 used immunohistochemistry (IHC) to determine CIP2A expression, the remaining 3 used reverse transcription-polymerase chain reaction (RT-PCR) to determine CIP2A expression. The 15 eligible studies provided 2348 samples in all regarding the relationship between CIP2A expression and solid tumor prognosis.

**Figure 4:**
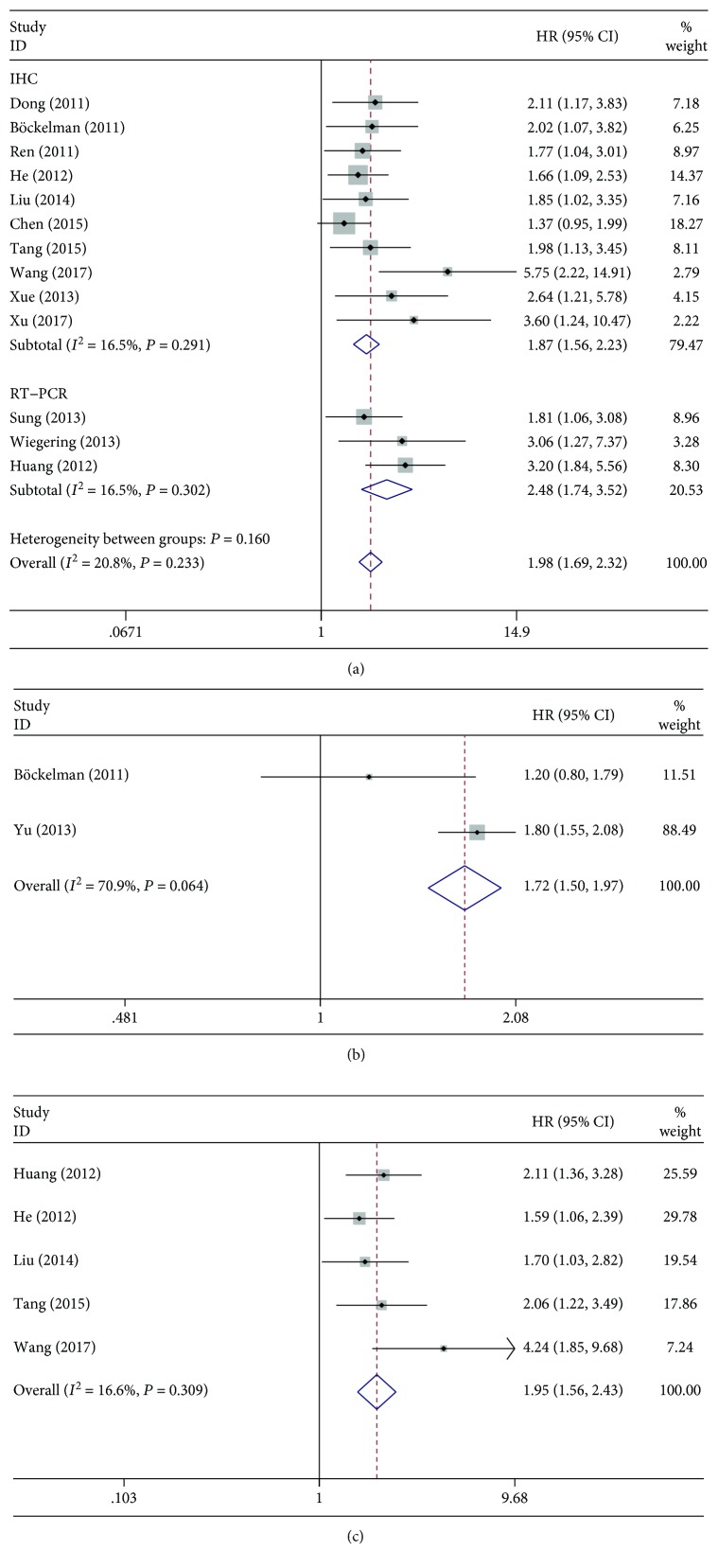
Forest plots of the meta-analysis of the association between CIP2A expression and the prognosis of patients with solid tumors. (a) Overall survival. (b) Disease-specific survival. (c) Time to tumor progression. Abbreviations: HR: hazard ratio; CI: confidence interval.

In this study, the unified prognostic parameter TTP was used instead of the outcome endpoints RFS and DFS which were similar in meaning. Therefore, the meta-analysis was based on 3 outcome endpoints (OS, DSS, and TTP). Overall, 13 studies were used in the meta-analysis for OS. The heterogeneity test reported a *P* value greater than 0.05; therefore, the fixed effects model was chosen to calculate the pooled HR and 95% CI. A significant correlation was observed between the expression of CIP2A and OS (pooled HR = 1.98, 95%CI = 1.69‐2.32) (Figure 4(a)). The stratified analysis based on the detection method further demonstrated that CIP2A expression was significantly associated with OS (Figure 4(a)) in both the IHC subgroup and the RT-PCR subgroup. Two studies were used in the meta-analysis for DSS. The heterogeneity test reported a *P* value of 0.064; therefore, the fixed effects model was used. Significant correlation between the expression of CIP2A and DSS was also detected (pooled HR = 1.72, 95%CI = 1.50‐1.97) (Figure 4(b)). Five studies were included in the meta-analysis for TTP. The fixed effects model was also used since the heterogeneity test reported a *P* value of 0.309. The results showed a significant correlation between the expression of CIP2A and TTP as well (pooled HR = 1.95, 95%CI = 1.56‐2.43) (Figure 4(c)). In summary, our meta-analysis suggests that the expression of CIP2A is predictive of OS, DSS, and TTP in solid cancer patients.

The results of Begg's funnel plot (see Supplementary [Supplementary-material supplementary-material-1]) and Egger's test indicated no publication bias for TTP (*P* = 0.053), but publication bias probably existed in the meta-analysis for OS (*P* < 0.01). In addition, a publication bias test could not be performed for DSS because of the insufficient study numbers.

## 4. Discussion

Esophagogastric junction adenocarcinoma (EGJA) has different clinicopathological features than distal gastric cancer. Although considerable progress of diagnosis and treatment has been made over the past few decades, the prognosis for patients with EGJA remains poor [36]. Previous studies have demonstrated a significant relationship between EGJA grade and prognosis. The prognosis of patients with well or moderate differentiation was better than that of patients with poor or no differentiation. Only patients with no lymph node metastasis had a significant correlation between tumor grade and survival when TNM staging was also added to the analysis, while the number of lymph nodes was an independent prognostic factor for EGJA [37]. Institutional variation and preoperative laboratory data such as nutritional status are also associated with EGJA prognosis [38, 39]. Biomarkers are also important indicators of tumor prognosis. However, few studies have reported that biomolecules are associated with EGJA prognosis, such as NFIB, an indicator of poor prognosis for EGJA [40]. In the present study, we investigated new biomarkers to further understand the pathological and molecular characteristics of EGJA and provide new targets for its diagnosis and treatment.

CIP2A is a well-known oncogene whose expression is closely related to the prognosis of various tumors. This study analyzed the expression pattern of CIP2A in the EGJA tissue microarray to determine its prognostic significance. The results demonstrated that CIP2A was highly expressed in EGJA compared to paracancers, and the Kaplan-Meier analysis revealed that the high expression of CIP2A indicated a poor prognosis for EGJA. Clinicopathological parameters such as age, grade, stage, and lymph node metastasis were also significantly correlated with the prognosis of EGJA, consistent with previous studies.

The combined application of the clinicopathological parameters and the CIP2A expression can help to further stratify EGJA patients for more accurate prediction. Multivariate Cox analysis showed that grade and CIP2A expression were independent prognostic factors for EGJA patients. Thus, we combined the grade with CIP2A expression, dividing the patients into high-risk, intermediate-risk, and low-risk groups. The Kaplan-Meier analysis of each group demonstrated that the combination of grade and CIP2A expression can predict patient survival more accurately, especially in the high-risk group with high grade and high expression of CIP2A indicating the worst prognosis. To compare the sensitivity and specificity of the survival prediction between grade and CIP2A expression, we performed a ROC analysis, in which a larger area under the ROC curve (AUC) is generally considered a better predictive model [41]. The results showed that grade and CIP2A expression had strong prognostic abilities, respectively. By combining these two predictors, further stratification can be used to obtain a more accurate prognosis and improve the accuracy and sensitivity of the prognosis of EGJA.

This retrospective study was designed to assess the prognostic power of the oncogene CIP2A in EGJA. The results suggested that CIP2A may be a new biomarker for the diagnosis and treatment of EGJA. However, because of the limitation of low case numbers (i.e., distant metastasis), the conclusion must be further validated in a large number of prospective clinical trials. Furthermore, we performed a meta-analysis to elucidate the relationship between CIP2A and the prognosis of solid tumors.

The meta-analysis results suggested that higher tumoral CIP2A expression is correlated with an unfavorable prognosis and is predictive of OS, DSS, and TTP in solid cancer patients. This conclusion is supported by the reported potential biological function of CIP2A. CIP2A is an inhibitor of the tumor suppressor protein phosphatase 2 and stabilizes MYC protein. It reportedly contributes to anchorage-independent cell growth and plays an important role in tumor formation [10]. Moreover, combined effects of CIP2A with other molecular and environmental factors probably exist and may differ among tumor types.

Because there are several limitations in our meta-analysis, the results need to be considered cautiously. First, the sample size used was not sufficient, particularly in the analysis of DSS. Second, heterogeneity existed due to the diverse CIP2A expression detection methods and the varied cutoff values chosen among studies. Third, there was no adjustment considering patients' characteristics like age and lifestyle. Furthermore, with the existing data, stratified meta-analysis based on tumor type could not be conducted. In order to draw a more convincing conclusion, analyses with larger sample size and adjusted individual data are needed, and subgroup analyses based on tumor type are also required. In conclusion, supported by a meta-analysis with a total of 15 studies (2348 patients total), this study indicated that CIP2A expression may be a predictive marker of OS, DSS, and TTP in solid cancer patients. This meta-analysis provides valuable information for the study of the relationship between CIP2A and solid cancer prognosis, although there are limitations.

## 5. Conclusion

This study investigated the clinical significance of CIP2A in EGJA by detecting the expression of CIP2A in EGJA and paracancer tissues and investigated the association between CIP2A and solid cancer prognosis in a systematic and statistic way. The results of this study suggest that CIP2A expression is an independent prognostic factor for EGJA patients and CIP2A may be a new biomarker for the diagnosis and treatment of EGJA. A meta-analysis further indicated that CIP2A expression may be a predictive marker of overall survival, disease-specific survival, and time to tumor progression in patients with solid tumors.

## Figures and Tables

**Table 1:** Correlations of CIP2A expression with clinicopathologic parameters in EGJA.

Variable	No. of patients	CIP2A expression		*P* ^a^
		Low (%)	High (%)	
Total cases	65	23 (35.4)	42 (64.6)	
Age (years)				0.140
≤65	24	11 (16.9)	13 (20.0)	
>65	41	12 (18.5)	29 (44.6)	
Gender				0.606
Male	51	18 (27.7)	33 (50.8)	
Female	14	5 (7.7)	9 (13.8)	
Grade				0.357
Low (I-II)	39	15 (23.1)	24 (36.9)	
High (III-IV)	26	8 (12.3)	18 (27.7)	
TNM stage				0.402
Early (I-II)	20	8 (12.3)	12 (18.5)	
Late (III-IV)	45	15 (23.1)	30 (46.2)	
Lymph metastasis				0.402
No	20	8 (12.3)	12 (18.5)	
Yes	45	15 (23.1)	30 (46.2)	
Metastasis				0.414
No	63	23 (35.4)	40 (61.5)	
Yes	2	0 (0.0)	2 (3.1)	

^a^Fisher's exact test.

**Table 2:** Cox regression analysis of risk factors associated with survival.

Prognostic variables	Survival	
	Hazard ratio (95% CI)	*P*
*Univariate analysis*		
Age	2.532 (1.192-5.378)	0.016^∗^
Gender	1.333 (0.628-2.827)	0.454
Grade	2.344 (1.226-4.481)	0.010^∗^
TNM stage	2.475 (1.083-5.657)	0.032^∗^
Lymph metastasis	2.492 (1.090-5.700)	0.031^∗^
Metastasis	5.054 (1.142-22.364)	0.033^∗^
CIP2A expression	2.273 (1.070-4.826)	0.033^∗^
*Multivariate analysis*		
Grade	2.399 (1.154-4.985)	0.019^∗^
CIP2A expression	2.881 (1.294-6.414)	0.010^∗^

^∗^ *P* < 0.05.

**Table 3:** Studies and data included in this meta-analysis.

Author	Year	Patients' country of origin	Cancer type	No. of patients	Stage/grade	Detection method	Percentage of CIP2A high expression, cutoff value	Median follow- up months	Outcome	Survival analysis method
Böckelman	2011	Finland	Tongue cancer	71	Grade I-III	IHC	32/71 (45.1%), score = 3	94.8	OS	M
Böckelman	2011	Finland	Serous ovarian cancer	524	I-IV	IHC	212/524 (40.5%), score = 2~3	105.6	DSS	M
Dong	2011	China	Non-small-cell lung cancer	90	I-IV	IHC	65/90 (72.2%), score ≥ 1	NA	OS	M
Ren	2011	China	Renal cell carcinoma	85	I-IV	IHC	36/85 (42.4%), staining intensity: 2-3	NA	OS	M
He	2012	China	Hepatocellular carcinoma	136	I-IV	IHC	85/136 (62.5%), 2+ to 3+	NA	OS, DFS	M
Huang	2012	China	Hepatocellular carcinoma	136	I-III	RT-PCR	68/136 (50.0%), above median mRNA expression levels	39	OS, RFS	M
Sung	2013	China (Taiwan)	Lung adenocarcinoma	98	I-III	RT-PCR	49/98 (50.0%), median value of mRNA expression levels in lung tumors	21.5	OS	M
Wiegering	2013	Germany	Colon cancer	104	I-IV	RT-PCR	NA, above median mRNA expression levels	NA	OS	M
Xue	2013	China	Bladder urothelial cell carcinoma	117	Ta, T1-T4	IHC	85/117 (72.6%), + to +++	57.1	OS	M
Yu	2013	China	Breast cancer	164	Grade I-III	IHC	58/164 (35.4%), 1+ or 2+	NA	DSS	M
Liu	2014	China	Nasopharyngeal carcinoma	280	I-IV	IHC	184/280 (65.7%), ROC curves	63.6	OS, DFS	M
Chen	2015	China (Taiwan)	Colorectal cancer	220	I-IV	IHC	91/220 (41.4%), H score ≥ 150	NA	OS	M
Tang	2015	China	Clear cell renal cell carcinoma	131	T1-T4	IHC	88/131 (67.2%), score ≥ 145	NA	OS, DFS	M
Wang	2017	China	Cervical cancer	127	I-IV	IHC	NA, + to +++	NA	OS, DFS	U
Xu	2017	China	Gallbladder carcinoma	65	I-IV	IHC	42/65 (64.6%), moderate or strong, 5-100% positive cells	NA	OS	M

IHC: immunohistochemistry; NA: not available; OS: overall survival; DSS: disease-specific survival; DFS: disease-free survival; RFS: recurrence-free survival; M: multivariate Cox proportional hazard regression; U: univariate survival analysis.

## Data Availability

The data used to support the findings of this study are available from the corresponding authors upon request.

## Supplementary Materials

Supplementary 1 Associations of clinicopathologic parameters and CIP2A expression with prognosis.

Supplementary 2 Begg's funnel plots for the studies involved in the meta-analysis of CIP2A expression and the prognosis of patients with solid tumors. (a) Overall survival. (b) Time to tumor progression. Abbreviations: loghr: logarithm of hazard ratios; s.e.: standard error.
