# Supplementary material for: Prognostic Significance of CIP2A in Esophagogastric Junction Adenocarcinoma: A Study of 65 Patients and a Meta-Analysis
Source: Dis Markers. 2019 Aug 22;2019:2312439. doi: 10.1155/2019/2312439 (PMC6724434; doi:10.1155/2019/2312439)
Supplement: Supplementary 1 — Associations of clinicopathologic parameters and CIP2A expression with prognosis. [file 2312439.f1.pdf]

Supplementary Material 1. Associations of clinicopathologic parameters and CIP2A expression with prognosis

Variable		No. of Patients (%)		P^{a}
		Survival	Death	
Total case		28(43.1)	37(56.9)	
Age(y)				0.015*
	≤65	15(23.1)	9(13.8)	
	>65	13(20.0)	28(43.1)	
Gender				0.377
	Male	23(35.4)	28(43.1)	
	Female	5(7.7)	9(13.8)	
Grade				0.028*
	Low (I-II)	21(32.3)	18(27.7)	
	High (III-IV)	7(10.8)	19(29.2)	
TNM Stage				0.018*
	Early (I-II)	13(20.0)	7(10.8)	
	Late (III-IV)	15(23.1)	30(46.2)	
Lymph Metastasis				0.018*
	No	13(20.0)	7(10.8)	
	Yes	15(23.1)	30(46.2)	
Metastasis				0.320
	No	28(43.1)	35(53.8)	
	Yes	0(0.0)	2(3.1)	
CIP2A Expression				0.030*
	Low	14(21.5)	9(13.8)	
	High	14(21.5)	28(43.1)	

[a] Fisher Exact test

* $P < 0.05$
