# Supplementary material for: Prognostic Significance of CIP2A in Esophagogastric Junction Adenocarcinoma: A Study of 65 Patients and a Meta-Analysis
Source: Dis Markers. 2019 Aug 22;2019:2312439. doi: 10.1155/2019/2312439 (PMC6724434; doi:10.1155/2019/2312439)

Supplementary Material 2. Begg's funnel plots for the studies involved in the meta-analysis of CIP2A expression and the prognosis of patients with solid tumors. **a.** Overall survival. **b.** Time to tumor progression. Abbreviations: loghr, logarithm of hazard ratios; s.e., standard error.

a

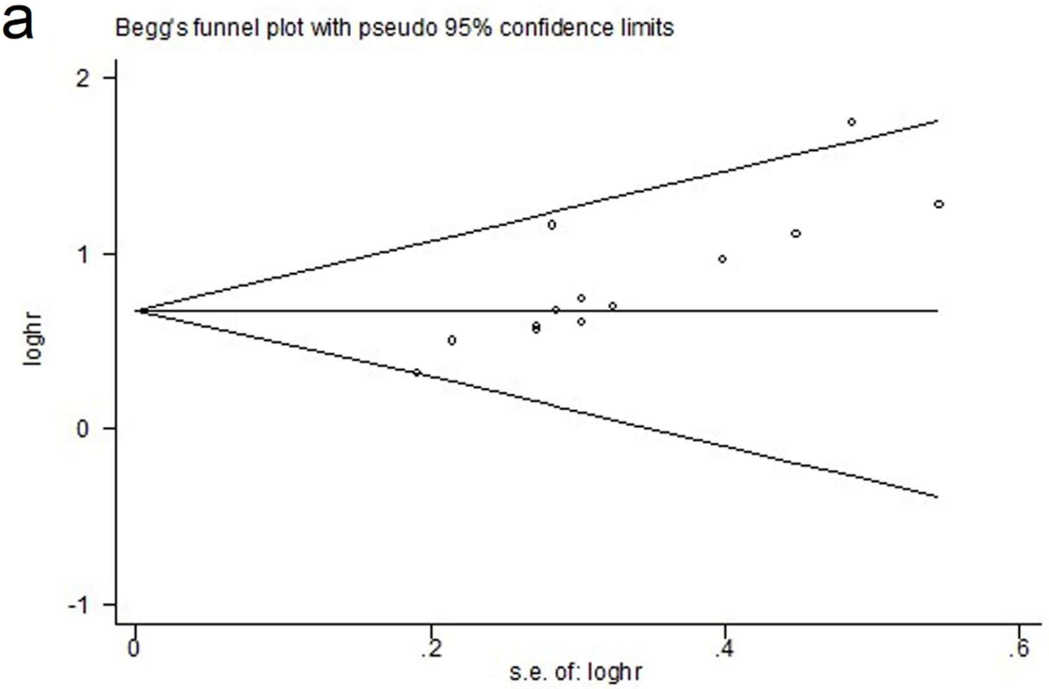

b

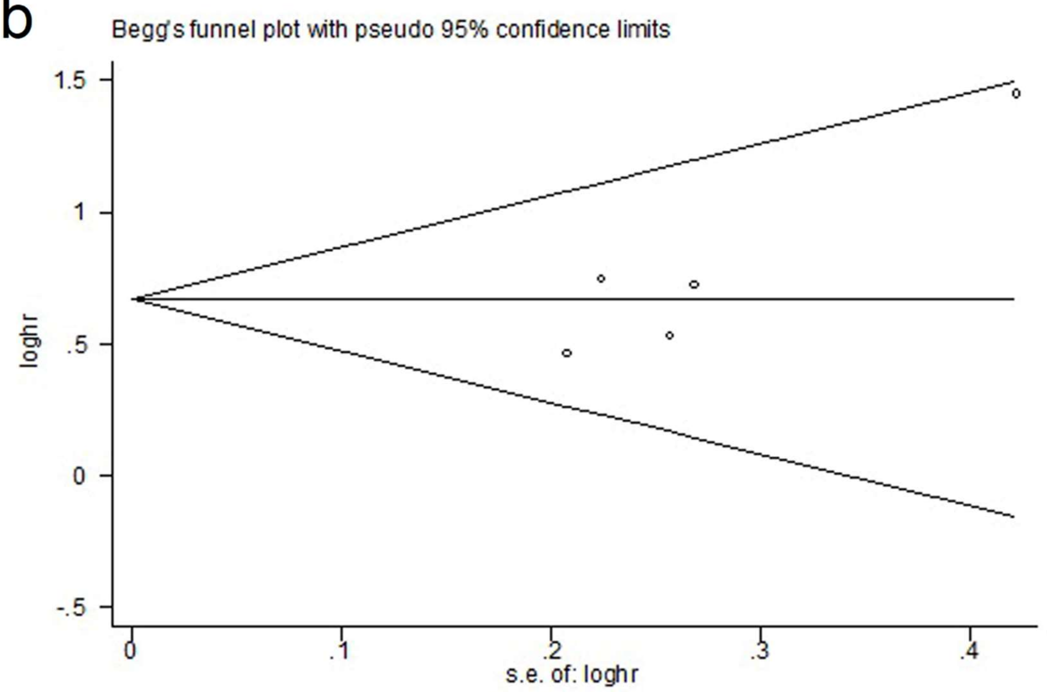

Supplement: Supplementary 2 — Begg's funnel plots for the studies involved in the meta-analysis of CIP2A expression and the prognosis of patients with solid tumors. (a) Overall survival. (b) Time to tumor progression. Abbreviations: loghr: logarithm of hazard ratios; s.e.: standard error. [file 2312439.f2.pdf]
